# Arginase-I enhances vascular endothelial inflammation and senescence through eNOS-uncoupling

**DOI:** 10.1186/s13104-017-2399-x

**Published:** 2017-02-02

**Authors:** Cuicui Zhu, Yi Yu, Jean-Pierre Montani, Xiu-Fen Ming, Zhihong Yang

**Affiliations:** 1 Cardiovascular and Aging Research, Division of Physiology, Department of Medicine, University of Fribourg, Chemin du Musée 5, 1700 Fribourg, Switzerland; 2 National Center of Competence in Research “Kidney.CH”, University of Zürich, Zürich, Switzerland

**Keywords:** Arginase-I, Endothelial cell, eNOS-uncoupling, Inflammation, Senescence

## Abstract

**Background:**

Augmented arginase-II (Arg-II) is implicated in endothelial senescence and inflammation through a mutual positive regulatory circuit with S6K1. This study was conducted to investigate whether Arg-I, another isoform of arginase that has been also reported to play a role in vascular endothelial dysfunction, promotes endothelial senescence through similar mechanisms.

**Results:**

The non-senescent human endothelial cells from umbilical veins (passage 2 to 4) were transduced with empty recombinant adenovirus vector (rAd/CMV) as control or rAd/CMV-Arg-I to overexpress Arg-I. Overexpressing Arg-I promoted eNOS-uncoupling, enhanced senescence markers including p53-S15, p21 and senescence-associated β-galactosidase (SA-β-gal) staining, and increased inflammatory vascular adhesion molecule-1 (VCAM-1) and intercellular adhesion molecule-1 (ICAM-1) as well as monocyte adhesion to endothelial cells without activating S6K1. All the effects of Arg-I were inhibited by the anti-oxidant N-acetylcysteine (NAC).

**Conclusions:**

Our study demonstrates that Arg-I promotes endothelial senescence and inflammatory responses through eNOS-uncoupling unrelated to activation of the S6K1 pathway.

## Background

Aging is a prominent risk factor for cardiovascular diseases [1]. Evidence has been presented that vascular aging and age-associated vascular diseases are attributable to endothelial senescence, an irreversible proliferation arrest with functional alterations [2–5]. Endothelial senescence or aging is characterized by reduced nitric oxide (NO) generation with concomitant augmented production of O_2_^.−^ resulting from endothelial NO synthase (eNOS)-uncoupling [2, 6–8], and enhanced inflammatory molecule expression such as VCAM-1 and ICAM-1 [3, 9]. This leads to enhanced adhesion and transmigration of monocytes into vascular wall, which facilitates the initiation and progression of atherosclerosis in aging [10, 11].

Among the various mechanisms of eNOS-uncoupling in aging and cardiovascular pathologies [8], augmented arginase activity in endothelial cells has been reported to cause eNOS-uncoupling through competing for their common substrate l-arginine [12]. Two isoforms of arginase encoded by different genes have been identified, i.e., Arg-I and Arg-II [13]. Previous studies including our own have shown that in human and murine vascular endothelial cells, Arg-II is the predominant isoenzyme [14–17], and inhibition of Arg-II improves endothelial function in mouse models of atherosclerosis, diabetes, and aging [14, 15, 18, 19].

Studies also report that Arg-I is the major isoform expressed in rat endothelial cells and contributes to impaired endothelial function in aging of this species [12, 20, 21]. Moreover, Arg-I is shown to be upregulated in the bone marrow stromal cells of diabetic mouse models, contributing to diabetes-associated osteoporosis [22]. A recent study also reports that Arg-I is upregulated in a mouse myocardial infarction model and in human aortic endothelial cells under ischemia through the transcription factor FoxO4, leading to decreased NO production and cardiac damage [23]. Interestingly, both Arg-I and Arg-II are upregulated in the perivascular adipose tissues of mice fed high-fat-diet, which is linked to eNOS-uncoupling in this tissue [24]. These studies suggest a role of both Arg-I and Arg-II in various aspects of cardiovascular disease depending on the models, species, and tissues of interest. There is evidence that Arg-I and Arg-II exert certain distinct biological functions, although they share the same enzymatic function in metabolizing l-arginine [25].

Therefore, it is important to investigate whether Arg-I and Arg-II share the same molecular mechanism in causing endothelial dysfunction. Recently, we provided evidence for a causal role of Arg-II in endothelial inflammatory responses and endothelial senescence through a mutual positive regulatory circuit with S6K1 [16]. However, it is unknown whether upregulation of Arg-I is capable of inducing aging-associated endothelial dysfunction. The main goal of our short study is therefore to investigate whether Arg-I plays a causal role in promoting endothelial cell senescence through similar mechanisms as Arg-II, i.e., through mTOR/S6K1 and/or eNOS-uncoupling.

## Methods

### Materials

All chemicals including those used for immunoblotting and anti-tubulin (T5168) antibody were obtained from Sigma (Buchs, Switzerland). Antibody against p21^Cip1^ (OP64) was purchased from Calbiochem (Genève, Switzerland); antibody against phosphor-p53-S15 (#9284s) was from Cell Signalling (Allschwil, Switzerland); antibodies against Arg-I (sc-18351), Arg-II (sc-20151) and p53 (sc-6243) were from Santa-Cruz (Nunningen, Switzerland); Alexa Fluor680-conjugated anti-mouse IgG (A21057); Carboxyfluorescein diacetate succinimidyl ester (CFDA-SE) and dihydroethidium (DHE) were from Molecular Probes/Invitrogen (Lucerne, Switzerland); IRDye800-conjugated anti-rabbit IgG (926-32211) were from LI-COR Biosciences (Bad Homburg, Germany); the membrane-permeable 4,5-Diaminofluorescein diacetate (DAF-2DA) was from VWR international SA (Dietikon, Switzerland); X-gal was from Promega (Dübendorf, Switzerland); Endothelial cell growth supplement (ECGS) pack was from PromoCell GmbH (Allschwil, Switzerland) and all cell culture media and materials were purchased from Gibco BRL (Lucerne, Switzerland).

### Generation of recombinant adenoviral (rAd)

Expression plasmids encoding a murine Arg-I (pCMV6-kan/neo-ArgI) was purchased from OriGene Technologies, Inc. Recombinant adenoviruses expressing the murine Arg-I (rAd/CMV-Arg-I) was carried out with the Gateway Technology (Invitrogen life Technologies) according to manufacturer’s instruction. The control empty rAd/CMV was from Invitrogen life Technologies.

### Endothelial cell culture and adenoviral transduction of the cells

The primary human umbilical vein endothelial cells (HUVEC) were generated from human umbilical cords. Isolation, culture and transduction of HUVEC by recombinant adenovirus were performed as previously described [2]. Human umbilical cords were obtained anonymously, following a prior informed consent, from healthy mothers after normal, full-term deliveries at the Daler Hospital, Fribourg, which does not require approval from a cantonal ethics committee according to the applicable laws, rules and regulations of the Swiss Association of Ethics Committees for research involving humans. The non-senescent cells of passage 2 to 4 (P2 to P4) were used for experiments. For experiments with NAC, NAC (5 mmol/L, pH 7.4) was added immediately after transduction until experiments were performed.

### Senescence-associated β-galactosidase (SA-β-gal) staining

SA-β-galactosidase staining was performed 7 days post transduction as described [2].

### Immunoblotting

Cell lysate preparation, SDS-PAGE, and immunoblotting, antibody incubation and signal detection were performed as described [26]. Quantification of the signals was performed using NIH Image 1.62 software.

### Detection of NO and superoxide level

NO and superoxide levels in cultured endothelial cells were assessed by staining the cells with fluorescent dyes DAF-2DA and DHE, respectively, as described previously [2].

### Monocyte adhesion to endothelial cells

The adhesion assay was performed as described previously [26]. Briefly, the human monocytic THP-1 cells were labeled with 5 μmol/L CFDA-SE in PBS at 37 °C for 8 min. The labeling was stopped with 1 ml of heat-inactivated FBS for 1 min. The labeled monocytes (4 × 10^5^ THP-1) were then added to the HUVECs that were transduced with recombinant adenoviruses and serum-starved for 12 h prior to the addition of labeled monocytes. After incubation for 15 min at 37 °C, the non-adherent THP-1 cells were washed twice with PBS and fixed in 2% paraformaldehyde. The images of adherent monocytes were captured under the fluorescent microscope (three different fields per sample were captured). The number of adherent monocytes was counted using the NIH ImageJ software (U. S. National Institutes of Health).

### Statistics

Data are given as mean ± SEM. In all experiments, n represents the number of independent experiments indicated in each figure. The Kolmogorov–Smirnov test was used to first determine whether the data deviated from Gaussian distributions. For normally distributed values, statistical analysis was performed with the Student *t* test for unpaired observations or analysis of variance (ANOVA) with Bonferroni’s post-test. For non-normally distributed values, nonparametric statistical analysis was performed with the Mann–Whitney test or the Kruskal–Wallis test with a Dunn’s multiple comparison post-test. p ≤ 0.05 was considered statistically significant.

## Results

### Overexpressing Arg-I in endothelial cells up-regulates adhesion molecule expression

**Fig. 1:**
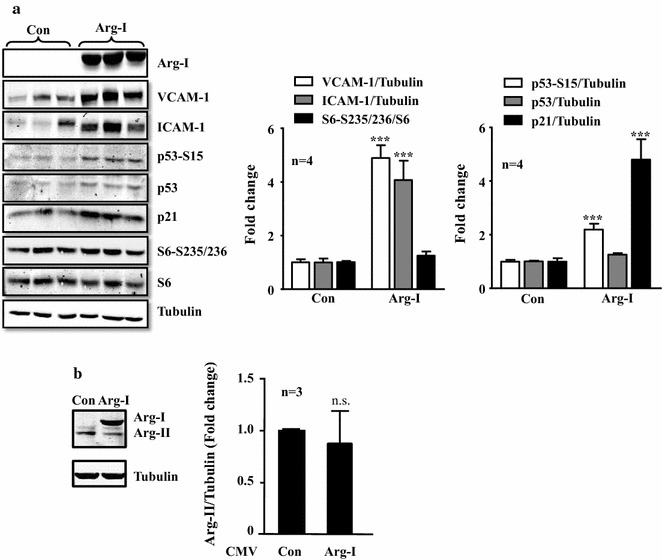
Overexpression of Arg-I promotes endothelial senescence and inflammation. The non-senescent endothelial cells (passage 2 to 4) were transduced with empty rAd/CMV vector as control (con) or rAd/CMV-Arg-I (Arg-I). Forty-eight hours post-transduction, the cells were serum-starved for 16 h, the cell lysates were then prepared and subjected to immunoblotting analysis of **a** Arg-I, expression of endothelial inflammation markers VCAM-1 and ICAM-1, and senescence markers p53-S15, p53 and CDK inhibitor p21^Cip1^ levels; **b** Arg-I and Arg-II expression. *Bar graphs* in the *right panels* show quantification of the signals. Tubulin served as loading control. ***p < 0.005 vs. control (con). *n.s.* not significant

In human endothelial umbilical vein cells, in which endogenous Arg-I expression is not detectable [14–16], adenovirus-mediated ectopic expression of Arg-I, as verified by immunoblotting (Fig. 1a), significantly enhanced expression levels of the inflammatory adhesion molecules i.e., VCAM-1 and ICAM-1 as well as the senescence markers, including p53-S15, p21^Cip1^ (Fig. 1a), and the number of SA-β-gal positive cells (see Fig. 3b in the later section). In contrast to the previously published study investigating the Arg-II isoenzyme [16], Arg-I did not activate mTOR/S6K1 signalling pathway as monitored by the phosphorylation status of its substrate S6 at S235/236 (Fig. 1a). To ensure that the effect of Arg-I overexpression was not due to the changes in Arg-II, the expression of Arg-II was monitored by immunoblotting. The Arg-II expression tended to be downregulated by Arg-I overexpression in the endothelial cells. However, this did not reach statistical significance (Fig. 1b), which rather excludes a role of Arg-II in Arg-I-induced endothelial senescence and dysfunction. These results provide evidence for a causal role of Arg-I in promoting endothelial senescence and senescence-associated inflammatory responses, which is independent of S6K1 activation.

### Overexpressing Arg-I in endothelial cells causes eNOS-uncoupling

**Fig. 2:**
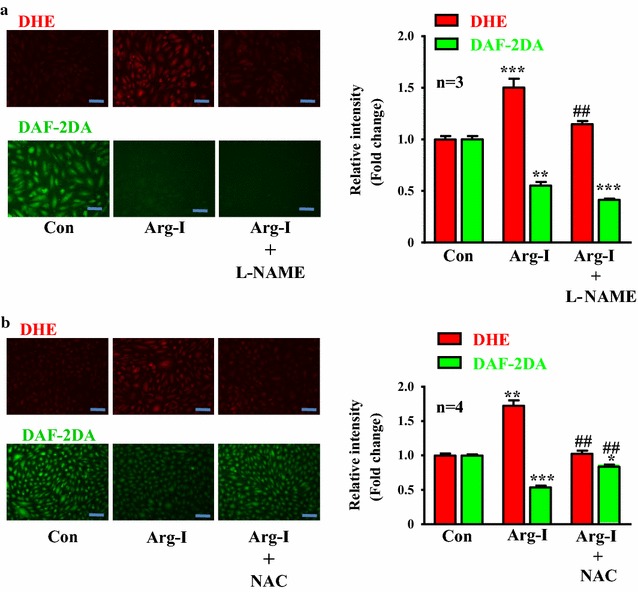
Overexpression of Arg-I causes eNOS uncoupling, which is prevented by antioxidant NAC. Cells were transduced as described in Fig. 1. Forty-eight hours post-transduction, the cells were serum-starved for 16 h and then subjected to DHE and DAF-2DA staining for detection of O_2_^.−^ (*red*) and NO (*green*), respectively. **a** Cells were treated with or without the eNOS inhibitor L-NAME (1 mmol/L, 1 h) during the last hour to demonstrate eNOS-uncoupling. **b** NAC (5 mmol/L) was added immediately after transduction and present in the culture medium until experiments were performed. *Bar graphs* in the corresponding *right panels* show quantification of the signals. *p < 0.05, **p < 0.01 and ***p < 0.005 vs. control (con); ^##^<0.01 and vs. Arg-I. *Scale bar*  0.2 mm

In endothelial cells, overexpression of Arg-I caused eNOS-uncoupling, i.e., impaired NO production (DAF-2DA staining) and enhanced intracellular superoxide generation (DHE staining) which was inhibited by the eNOS inhibitor L-NAME (1 mmol/L, 1 h, Fig. 2a). This result demonstrates a causal role of Arg-I in eNOS-uncoupling. Moreover, treatment of the cells with anti-oxidant NAC (5 mmol/L) prevented eNOS-uncoupling evoked by Arg-I, i.e., inhibition of superoxide generation (DHE signal) and enhanced bioavailability of NO (DAF-2DA signal), demonstrating re-coupling of eNOS by the drug (Fig. 2b).

### Recoupling of eNOS prevents Arg-I-induced endothelial inflammation and senescence

**Fig. 3:**
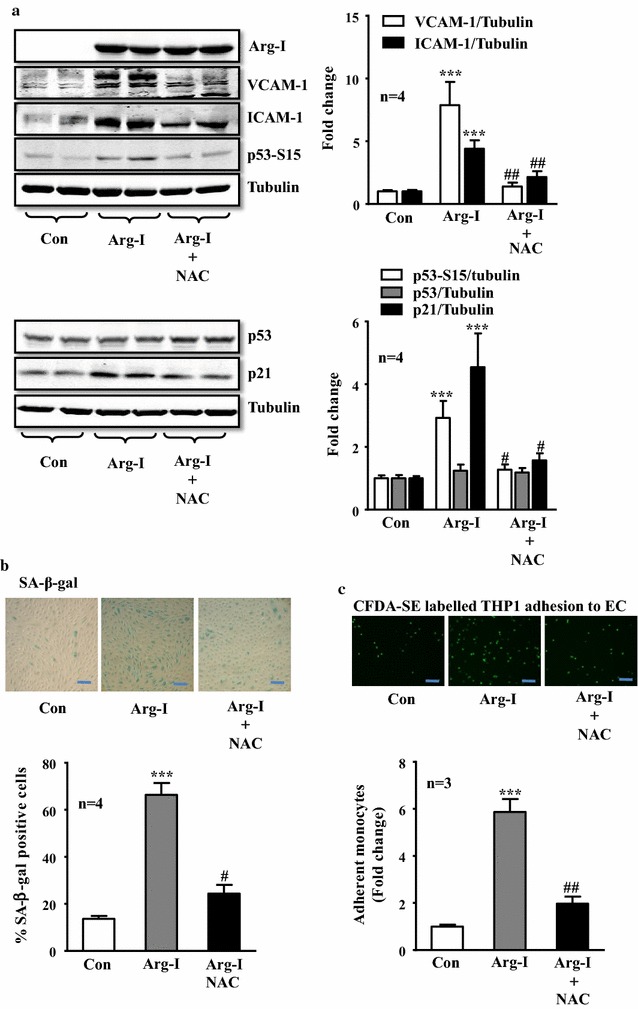
The antioxidant NAC significantly blunted senescence and inflammation caused by Arg-I overexpression in endothelial cells. Non-senescent endothelial cells were transduced and treated with NAC as described in Fig. 2b. **a** Immunoblotting analysis of endothelial inflammation markers VCAM-1 and ICAM-1 expression, and senescence markers p53-S15 and p21^Cip1^ levels. Tubulin served as loading control. Lysates were prepared 64 h post-transduction with serum-starvation for the last 16 h. **b** SA-β-gal staining on day 5 post-transduction. *Bar graphs* show quantification of relative of SA-β-gal positive cells. **c** Monocytes adhesion assay. Arg-I overexpressing HUVECs were treated with or without NAC (5 mmol/L, 64 h) after transduction. CFDA-SE fluorescence labeled THP-1 monocytes were then added to HUVECs. After washing, adhesion of the labeled monocytes to endothelial cells was evaluated. *Bar graphs* in the *right* or *lower panels* show quantification of the corresponding signals. ***p < 0.005 vs. control (con). ^#^p < 0.05, ^##^p < 0.01 vs. Arg-I. *Scale bar*  0.2 mm

We then further investigated if recoupling of eNOS function is able to prevent senescence-promoting effects of Arg-I. For this purpose, NAC (5 mmol/L) was added to cells overexpressing Arg-I in young endothelial cells to recouple eNOS function as show in Fig. 2b. Cellular senescence and inflammatory responses caused by Arg-I gene overexpression, (i.e., enhanced VCAM-1 and ICAM-1 expression, elevated p53-S15 and p21^Cip1^ protein levels, and increased number of positively stained cells for SA-β-gal), were all prevented by NAC (Fig. 3a, b). In accordance, adhesion of THP-1 monocytes on endothelial cells was significantly enhanced in the cells with Arg-I gene overexpression, which was inhibited by NAC (Fig. 3c). These results demonstrate that eNOS-uncoupling is not only associated with endothelial senescence, but also mediates endothelial senescence and senescence-associated inflammation caused by Arg-I.

## Discussion

Endothelial senescence phenotypes including eNOS dysfunction and inflammatory activation are considered to promote age-associated progression of vascular diseases [10, 11, 27]. Enhanced expression and activity of arginase including both Arg-I and Arg-II isoforms have been shown to play a role in vascular aging [12, 15, 16, 19, 21, 28]. Our recent published study provides additional evidence that Arg-II isoenzyme is not only involved in eNOS-uncoupling, but also plays a causal role in promoting endothelial aging [16]. Moreover, we uncovered a novel mechanism by which Arg-II promotes endothelial senescence, i.e. through a positive feedback loop with the S6K1 pathway, an important player in cellular and organism aging [16]. The isoenzyme Arg-I, which was originally identified as a constitutively-expressed enzyme in hepatocytes, is also inducible in other cells/tissues including macrophages, endothelial cells in the heart of mice or endothelial cells of rats, bone marrows, and perivascular adipose tissues [23, 24, 26]. Although both isoenzymes share the same enzymatic function in metabolizing l-arginine, there is evidence that Arg-I and Arg-II exert certain distinct biological functions [25]. In the current study, we demonstrate that Arg-I, once overexpressed in vascular endothelial cells, similar to Arg-II, also plays a causal role in promoting endothelial senescence. However, this effect of Arg-I is independent on a crosstalk with S6K1 signalling.

Previous studies, including our own, have shown that in human and murine endothelial cells, Arg-II is the predominant isoenzyme [14–17]. Our studies on the roles of arginase in various pathologic cardiovascular processes have thus far focused on Arg-II because of the nature of our working system, i.e. human endothelial cells and mice as animal model [14, 16, 26, 29–33]. Despite the difference in their expression pattern in various species, the role of Arg-I and Arg-II in eNOS dysfunction and aging seems the same [12, 15, 19, 21, 28], suggesting that they exert their physiological or pathophysiological functions through similar mechanism(s). However, direct evidence remains to be provided. In the current study, by overexpressing Arg-I in HUVECs which do not express detectable level of Arg-I under basal conditions [14–16], we demonstrate that Arg-I, once expressed in the cells, also plays a causal role in induction of endothelial senescence phenotype including increased senescence markers, eNOS-uncoupling, elevated adhesion molecule expression, enhanced monocyte-endothelial cell interaction. Similar to Arg-II, Arg-I-induced endothelial senescence phenotype is attributable to eNOS-uncoupling, since the recoupling of eNOS by anti-oxidant NAC blunted the effect of Arg-I. However, Arg-I does not share all the functions of Arg-II. We have recently provided evidence showing that a mutual positive crosstalk between S6K1 and Arg-II causes eNOS-uncoupling, leading to acceleration of vascular endothelial aging [16]. In contrast to Arg-II [16, 32], we did not observe S6K1 activation by overexpressing Arg-I in the same type of the cells, suggesting that S6K1 is not necessarily the mediator of Arg-I-induced eNOS-uncoupling and cellular senescence. Moreover, we found no significant increase of Arg-II (rather a tendency toward a decrease) in the cells overexpressing Arg-I. These results exclude the possibility that the effect of Arg-I is mediated through Arg-II.

## Conclusion

Taken together, our study provides evidence for a causal role of Arg-I in promoting endothelial senescence, confirming that both Arg-I and Arg-II, once upregulated, have similar functions to induce eNOS-uncoupling and accelerate endothelial senescence. In contrast to Arg-II, the effect of Arg-I does not involve activation of S6K1 signaling. The molecular basis for this subtle difference between Arg-I and Arg-II awaits further investigation.

## Abbreviations

Arg-I: arginase-I
Arg-II: arginase II
CFDA-SE: carboxyfluorescein diacetate succinimidyl ester
CMV: cytomegalovirus
DAF-2DA: 4,5-Diaminofluorescein diacetate
DHE: dihydroethidium
ECGS: endothelial cell growth supplement
eNOS: endothelial nitric oxide synthase
HUVECs: umbilical vein endothelial cells
ICAM-1: intercellular adhesion molecule-1
L-NAME: L-N^G^-Nitroarginine methyl ester
NAC: N-acetylcysteine
rAd: recombinant adenovirus
S6K1: p70 ribosomal protein S6 kinase 1
SA-β-gal: senescence-associated β-galactosidase
VCAM-1: vascular adhesion molecule-1
